# Direct-acting antiviral agents in the therapy of hepatitis C virus-related mixed cryoglobulinaemia: a single-centre experience

**DOI:** 10.1186/s13075-017-1280-6

**Published:** 2017-04-08

**Authors:** Gianfranco Lauletta, Sabino Russi, Fabio Pavone, Angelo Vacca, Franco Dammacco

**Affiliations:** Department of Biomedical Sciences and Human Oncology, University of Bari “Aldo Moro”, Piazza G. Cesare, 11-70124, Bari, Italy

**Keywords:** Cryoglobulinaemia, Direct-acting antiviral agents, Hepatitis C virus, Rheumatoid factor

## Abstract

**Background:**

The efficacy and safety of direct-acting antiviral agents (DAAs) were evaluated in a cohort of prospectively enrolled patients with hepatitis C virus (HCV)-related mixed cryoglobulinaemia (MC), an immune complex-mediated vasculitis of small and medium vessels in which the pathogenetic role of HCV has been clearly established.

**Methods:**

Twenty-two patients received DAAs. Clinical and laboratory features were recorded at baseline, every 4 weeks until the end of treatment (EoT), and 12 weeks afterwards. Primary efficacy endpoints were (a) sustained virological response 12 weeks after therapy completion (SVR12), (b) regression of symptomatology (clinical response) and (c) cryoglobulin disappearance or cryocrit reduction ≥50% (immunological response). Complete response (CR) was defined as the occurrence of all three primary endpoints; partial response (PR) was defined as the occurrence of SVR12, with or without either immunological or clinical response; and no response was defined as missing the achievement of all three endpoints.

**Results:**

All patients reached SVR12. Compared with basal values, mean cryocrit values were significantly decreased at EoT and SVR12. A significant reduction of alanine transaminase and a parallel increase of complement component C4 levels were also detected. Rheumatoid factor activity was significantly reduced at EoT but not at SVR12. At SVR12, a CR was established in 14 patients (63.7%) and a PR in 8 patients (36.3%). In one patient with small lymphocytic lymphoma, the tumour progressed despite viral clearance. Mild adverse events were recorded in nine patients (40.9%).

**Conclusions:**

The response rates induced by the use of DAAs in patients with MC were remarkably higher than those previously achieved with pegylated interferon-α/ribavirin, with or without rituximab. A much longer follow-up is desirable to achieve useful information in terms of persistent viral clearance and clinical response.

## Background

Hepatitis C virus (HCV) is a major cause of chronic liver disease that can potentially progress to cirrhosis and hepatocellular carcinoma (HCC) [1–3]. It has been estimated that about 130 million to 150 million people worldwide have HCV infection and that HCV causes about 500,000 deaths each year [4]. In addition to liver damage, HCV infection is associated with several extrahepatic disorders [5]; among them, the striking association between HCV infection and mixed cryoglobulinaemia (MC), an immune complex-mediated vasculitis of small and medium vessels, has been clearly established [6, 7]. On the basis of B-cell clonal expansion that can be detected in the majority of patients, MC can also be considered a low-grade, indolent lymphoproliferative disorder [8].

Clinically, MC is characterised by the classic Meltzer’s triad of symptoms (purpura, weakness and arthralgia). The clinical spectrum of MC is quite variable, however, ranging from skin lesions, including recurrent purpuric eruptions, leg dyschromatosis and torpid ulcers, to peripheral neuropathy and renal damage. In a minority of patients, multi-organ involvement may result in life-threatening conditions [9, 10]. In our experience, compared with patients with chronic HCV infection without cryoglobulinaemia, HCV-positive patients with MC exhibit a lower rate of liver fibrosis and a lower cumulative probability of developing cirrhosis and HCC, but a more frequent occurrence of renal failure, neurologic impairment and B-cell non-Hodgkin lymphoma (B-NHL). Even so, the 15-year survival rates in these groups have been found to be roughly similar [11].

The therapeutic approach to MC has conventionally been based on the use of steroids and immunosuppressive drugs. Interferon (IFN)-based treatment was introduced in 1987, before the identification of HCV as the major etiologic factor [12, 13]. Subsequently, the antiviral combination of pegylated interferon-α plus ribavirin (pIFN-α + RBV) was employed to obtain a sustained virological response (SVR), and for many years, this combination has been considered the standard of care for the treatment of chronic HCV infection, capable of inducing SVR and clinical remission of vasculitis in about 78% of patients with MC [14, 15]. A multicentre, open-label study showed viral response rates ranging from 36% to 64% according to viral genotype and a clinical response in 88% of the patients [16]. The association of rituximab (RTX), an anti-CD20 monoclonal antibody aimed at inducing an effective B-cell depletion, resulted in further benefits, both in patients refractory to antiviral therapy [17, 18] and in those previously untreated [19]. In our experience, the addition of RTX to the pIFN-α + RBV combination has resulted in a 33% complete response (CR) (i.e., virological, immunological and molecular response) [19].

However, the efficacy of therapy in terms of viral clearance has remained unsatisfactory and is burdened with remarkable side effects. The use of first-generation protease inhibitors, such as boceprevir or telaprevir, associated with pIFN-α + RBV in a triple-combination therapeutic regimen resulted in significantly higher response rates in patients with chronic HCV infection without and with MC. Serious adverse events were recorded in almost 50% of patients, however [20].

The introduction of all-oral, direct-acting antiviral agents (DAAs) has dramatically changed the treatment of chronic HCV and consequently the prognosis of these patients. According to the viral molecular target, DAAs include inhibitors of non-structural proteins NS3/NS4 (simeprevir, paritaprevir and grazoprevir), NS5A (ledipasvir [LDV], daclatasvir [DCV], ombitasvir, elbasvir and velpatasvir) and NS5B (sofosbuvir [SOF] and dasabuvir) that are already available in Italy, except elbasvir, grazoprevir and velpatasvir, for which approval is pending. These drugs have been shown to be capable of eradicating the infection in over 90% of patients with chronic HCV, with limited variations related to viral genotype [21]. An additional advantage of DAAs is the possibility of avoiding IFN-related side effects. However, owing to the high costs of these drugs, it has been necessary to fix priority criteria among patients to be treated. Both the American and the European associations for the study of liver diseases, as well as the Italian Medicines Agency (AIFA), have established that extrahepatic manifestations of HCV infection, such as cryoglobulinaemic vasculitis and lymphoproliferative disorders, should be recognised as priority indications for treatment with DAAs [22, 23].

So far, only a few papers have been published on the efficacy and safety of the use of DAAs in patients with MC. Saadoun et al. [24] reported a 74% rate of sustained virological response 12 weeks after therapy completion (SVR12) and an 87.5% clinical response rate in 24 patients with HCV-related MC treated with an SOF plus RBV combination. In another study, carried out with 12 patients with MC (7 of whom had renal involvement) treated with SOF-based regimens, the SVR12 rate was 83% and was associated with improvement in serum creatinine and reduction in proteinuria in patients with glomerulonephritis. In addition, an overall reduction or disappearance of cryoglobulins was recorded in 89% of the patients [25]. Mention should also be made of a prospective double-centre (Florence/Rome) study of a cohort of 44 consecutive patients with HCV-associated MC, 2 of whom had evolved into an indolent NHL with monoclonal B-cell lymphocytosis. Following guideline-tailored therapy with DAAs, an SVR was demonstrated 12 and 24 weeks post-treatment in parallel with clinical response in all patients. A striking reduction in the mean cryocrit value was revealed at SVR12, and an even greater reduction was observed for sustained virological response 24 weeks after therapy completion (SVR24). A partial vasculitis response and a roughly 50% reduction of cryocrit were detected in the two patients with lymphoma. Adverse events were rather frequent but usually mild [26]. More recently, Bonacci et al. [27] reported a large prospective study of 64 cryoglobulinaemic patients with HCV infection, divided into those with symptomatic (55% of cases) cryoglobulinaemia and those with asymptomatic cryoglobulinaemia (45% of cases). Sixteen percent of the patients received an IFN-based DAA combination, whereas IFN-free regimens were administered to the remaining patients. Overall, 94% of patients showed SVR12, and 71% achieved a clinical CR. In 10 of 13 patients, concomitant immunosuppressive therapy was reduced or withdrawn. Forty-eight percent of patients achieved an immunological response, defined as disappearance of circulating cryoglobulins and normalisation of complement and/or rheumatoid factor (RF). However, despite SVR12 in 52% of patients, cryoglobulins or complement consumption or RF activity persisted, a low baseline cryocrit level being the only factor associated with an immunological CR.

We report the results of our single-centre, prospective study of a cohort of patients with MC whose main features can be summarised as follows: (a) unresponsiveness to or relapse after the previous standard-of-care treatment consisting of pIFN-α + RBV combination, or previously untreated patients; (b) the assignment to receive variable combinations of DAAs according to the guidelines of the AIFA eligibility criteria; and (c) a post-treatment evaluation at weeks 12 and 24, with a prolonged follow-up until 12 months in some cases.

## Methods

### Patients

Twenty-two consecutive HCV-positive patients with MC were enrolled in this study, with the aim being to assess both the efficacy and the safety of all-oral, IFN-free DAAs. Thirteen patients were non-responsive to or relapsed after previous pIFN-α + RBV combination therapy, whereas the remaining nine patients were treatment-naïve. Written informed consent was obtained from all patients. Because treatments were administered on-label, no approval by the ethics committee was required, and the study was conducted according to the principles of the Declaration of Helsinki.

### Clinical and virological evaluation

All patients were thoroughly examined according to validated criteria reported elsewhere [28]. Clinical features included the presence of purpura, fatigue and arthralgia, associated or not with skin ulcers. Renal damage and/or peripheral nervous system involvement were also looked for. Circulating cryoglobulins were detected as previously described [29–31] and immunochemically characterised by immunofixation, and their amounts were quantified as cryocrit (%). Serum HCV RNA levels were assessed using the AmpliTaq real-time polymerase chain reaction system with a detection threshold of 15 IU/ml (Roche Molecular Systems Inc., Pleasanton, CA, USA), whereas HCV genotyping was performed using the VERSANT HCV genotype 2.0 assay (LiPA) (Siemens Healthcare Molecular Diagnostics, Berkeley, CA, USA).

### Treatment schedules

Patients admitted to treatment had previously been examined by transient elastography (FibroScan; Echosens, Paris, France) for the assessment of liver fibrosis grade. Antiviral therapy was prescribed according to the AIFA eligibility criteria, HCV genotype, grade of liver fibrosis and prior treatment experience. For patients with RBV treatment, dose reduction was considered in accordance with technical schedule. The following regimens were employed: (a) daily SOF (400 mg) plus RBV (1000 to 1200 mg, depending on whether the patient’s body weight was ≤75 or >75 kg); (b) ombitasvir/paritaprevir/ritonavir (12.5 mg/75 mg/50 mg) plus dasabuvir (250 mg) (3D combo) twice daily, with or without weight-based RBV; (c) SOF/LDV (400 mg/90 mg) once daily; and (d) SOF (400 mg) plus DCV (60 mg) (SOF/DCV) once daily.

### Biochemical evaluation

In addition to clinical examination, baseline evaluation included aspartate transaminase/alanine transaminase (ALT) values, haemoglobin level, platelet count, RF activity, serum levels of C3 and C4 complement component fractions, and serum protein electrophoresis. The same parameters were evaluated every 4 weeks during treatment and 12 weeks after therapy completion to establish whether SVR12 had been achieved.

### Endpoints

Primary efficacy endpoints were (a) SVR12 (virological response), (b) regression of clinical manifestations of vasculitis (clinical response) and (c) cryocrit reduction by at least 50% or disappearance of cryoglobulins (immunological response). CR was defined as the occurrence of all primary endpoints, partial response (PR) as the occurrence of SVR12 with either immunological or clinical response, and no response (NR) as the lack of all criteria.

### Statistical analyses

To assess the statistical power and significance of CR proportion (π_1_), criterion power analysis was performed using G*Power 3.1 (Heinrich Heine University, Düsseldorf, Germany) with a one-tailed binomial test (one-sample case), assuming as the constant proportion the frequency of CR to pIFN-α + RBV therapy in patients with cryoglobulinaemia (π_0_ = 0.33) [19]. Sensibility (statistical power) was fixed at 0.80. This was a one-tailed test because we assumed a greater antiviral efficacy of DAAs as compared with pIFN-α + RBV therapy. To evaluate the frequencies and differences of virological and immunological responses, as well as C4 complement fraction, RF and ALT level normalisation, continuous variables were categorised as dichotomous variables and statistically analysed by cross-tabs and χ^2^ test.

Continuous variables were first subjected to descriptive statistical analyses to verify the normality of data (Kolmogorov-Smirnov test). The statistical significance of variable changes across the study was assessed by a general linear model (GLM) repeated measures analysis. Three factors (baseline, end of treatment [EoT] and SVR12 time points) and four variables (cryocrit, RF, C4 complement fraction and ALT levels) were taken into consideration. HCV RNA serum levels were excluded from the GLM because the test results for HCV were negative in all patients after treatment. The Huynh-Feldt test and group comparisons by simple contrasts were used for univariate repeated measures analysis of variance to evaluate EoT and SVR12 time point variations against the baseline. Two-tailed α probability (*p* value), estimation of the effect size in the sample (η^2^), and statistical power were reported, with *p* < 0.05 being considered significant. Statistical analyses were performed by using IBM SPSS Statistics version 20.0 software (IBM, Armonk, NY, USA).

## Results

Fourteen patients were treated with the SOF/RBV regimen for 12–24 weeks according to viral genotype and fibrosis stage, and seven patients with genotype 1 infection were treated with such a regimen before the availability of more effective combinations that all guidelines considered as optimal. Three patients received a 3D combo regimen, four with SOF/LDV and one SOF/DCV combination. Thirteen of twenty-two patients had experienced treatment failure in previous IFN-based therapeutic regimens. No statistically significant relationship was found between previous treatment failure and response to DAAs.

Table 1 summarises the main clinical, biochemical and virological characteristics of the patients enrolled. The subjects’ mean age was 66.9 years, ranging from 46 to 84, and the male/female ratio was 8/14. All patients were viraemic with HCV RNA levels ranging from 2.1 to 7.7 logarithmic IU/ml. The mean cryocrit value was 1.8%, ranging from 0.5% to 4%. ALT levels ranged from 13 to 668 IU/L (mean ± SD 104.8 ± 144.7), and C4 complement fraction ranged from 0 to 24 mg/dl (mean ± SD 9.6 ± 7.3). RF activity ranged from 0 to 530 IU/ml (mean ± SD 69.3 ± 119.1). Cryoglobulins were immunochemically characterised as type 2 MC (monoclonal immunoglobulin M [IgM]/polyclonal IgG) in 21 patients and as type 3 MC (polyclonal IgM/polyclonal IgG) in the remaining 1 patient.

**Table 1:** Baseline clinical, virologic and laboratory parameters of 22 patients with chronic hepatitis C virus infection with mixed cryoglobulinaemia

Parameters	Data
Age, years, mean ± SD (range)	66.9 ± 11.2 (46–84)
Female sex, *n* (%)	14 (63.6)
Serum HCV RNA, logarithmic IU/ml, mean ± SD	6.02 ± 1.2 (2.1–7.7)
HCV genotype, *n* (%)	
GT1	14 (63.6)
GT2	7 (31.8)
GT3	1 (4.6)
Cryocrit, %, mean ± SD (range)	1.8 ± 1.3 (0.5–4)
Cryoglobulin type, *n* (%)	
II	21 (95.4)
III	1 (4.6)
C3, mg/dl (n.v. 90–180) (mean ± SD)	93.4 ± 15.3 (51–118)
C4, mg/dl (n.v. 10–40) (mean ± SD)	9.6 ± 7.3 (0–24)
RF, IU/ml (n.v. 10–15) (mean ± SD)	69.3 ± 119.1 (0–530)
Clinical features, *n* (%)	
Meltzer’s triad	22 (100)
Glomerulonephritis	4 (18.1)
Peripheral neuropathy	2 (9.1)
Liver involvement	
ALT, IU/L (n.v. 12–78) (mean ± SD)	104.8 ± 144.7 (13–668)
Cirrhosis, *n* (%)	12 (54.5)
HCV-related tumours, *n* (%)	
Hepatocellular carcinoma	2 (10)
Non-Hodgkin lymphoma	1 (5)
Small lymphocytic lymphoma	1 (5)

*Abbreviations: ALT* Alanine transaminase, *C3* and *C4* Complement components, *GT* Genotype, *HCV* Hepatitis C virus, *RF* Rheumatoid factor, *n.v.* Normal values

Meltzer’s triad of symptoms (purpura, arthralgia and weakness) was consistently present in all patients. Cryoglobulinaemic glomerulonephritis was recorded in four patients (18.2%) and severe peripheral neuropathy in two patients (9.1%). Autoimmune haemolytic anaemia and idiopathic thrombocytopenic purpura were diagnosed in two patients each. In addition, four patients (18.2%) showed a monoclonal IgG component (two with κ light chains and two with λ light chains) on serum protein electrophoresis, and three patients received a diagnosis of NHL, one with stage 4 small lymphocytic lymphoma (SLL) who received SOF/RBV therapy for 24 weeks followed by four RTX infusions at 1-week intervals, one with marginal zone B-NHL that extended to ocular adnexa previously treated with pIFN-α + RBV + RTX, and one with diffuse centrocytic B-NHL who had received chemotherapy 2 years before that had resulted in CR. Finally, two patients who had previously received a diagnosis of HCC were treated with locoregional ablation therapies with no evidence of recurrence on the basis of contrast-enhanced ultrasound and computed tomography, both before starting therapy and following its completion. At FibroScan evaluation, 12 patients showed advanced liver fibrosis (6 with F3 and 6 with F4 fibrosis and no signs of decompensation), whereas an F1 or F2 score was recorded in the remaining 10 patients.

**Fig. 1:**
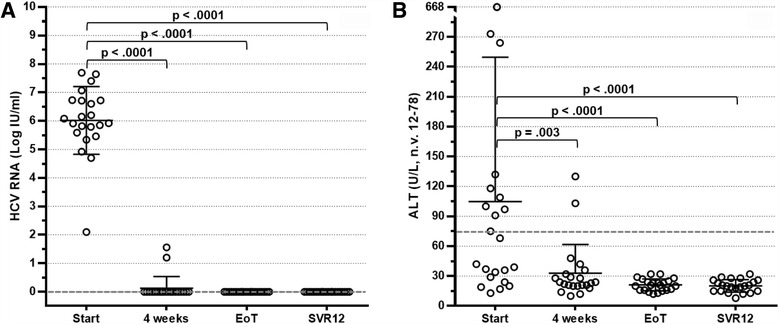
Distribution of hepatitis C virus (HCV) RNA (**a**) and alanine transaminase (ALT) (**b**) levels in 22 HCV-positive patients with cryoglobulinaemia treated with direct-acting antiviral agents at baseline, at week 4, at the end of treatment (EoT) and 12 weeks after treatment. *SVR12* Sustained virological response 12 weeks after therapy completion

Four weeks after starting therapy, HCV RNA determination was found to be almost invariably below the lower limit of detection. A rapid and progressive reduction of ALT levels until normalisation was also observed. These strikingly positive results were confirmed throughout the length of treatment. Consequently, all patients reached an EoT response and SVR12 (Fig. 1a) in step with persistent ALT normalisation (Fig. 1b). Interestingly, seven patients with genotype 1 infection who were given suboptimal SOF/RBV combination therapy also showed SVR12.

**Fig. 2:**
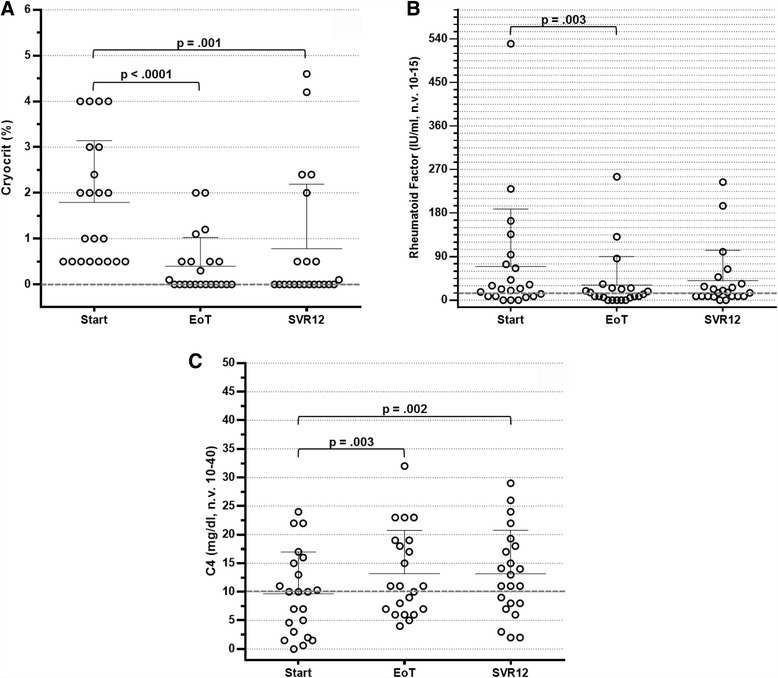
Distribution of cryocrit levels (**a**), rheumatoid factor activity (**b**) and complement component C4 levels (**c**) in 22 hepatitis C virus-positive patients with cryoglobulinaemia treated with direct-acting antiviral agents at baseline, at week 4, at the end of treatment (EoT) and 12 weeks after treatment. *SVR12* Sustained virological response 12 weeks after therapy completion

An immunological response was observed at EoT in 18 patients (81.8%). In 12 patients (54.5%), cryoglobulins completely disappeared, whereas in 6 patients (27.3%), a cryocrit reduction ≥50% of basal values was observed (Fig. 2a). A less remarkable reduction in cryocrit was calculated in two additional patients, and roughly unchanged levels were observed in the remaining two patients. At the time of SVR12, an overall immunological response was recorded in 17 patients (77.3%). More precisely, cryoglobulins were undetectable in 13 of them and were reduced to <50% of basal value in 4 patients (Fig. 2a). Conversely, cryoglobulins were unchanged in three of the remaining five patients at SVR12, and two patients had relapsed at EoT evaluation.

Additional immunological features also showed a remarkable improvement. As shown in Fig. 2b, test results for RF activity were negative in 12 patients at EoT (54.5%) and in 11 patients at SVR12 (50%). In two patients, RF levels were undetectable at EoT but were increased again at SVR12, whereas in one patient the RF test result was negative only at SVR12. Serum C4 levels increased to the normal range in 10 patients at EoT (45.5%) and in 13 patients at SVR12 (59.1%) (Fig. 2c).

**Fig. 3:**
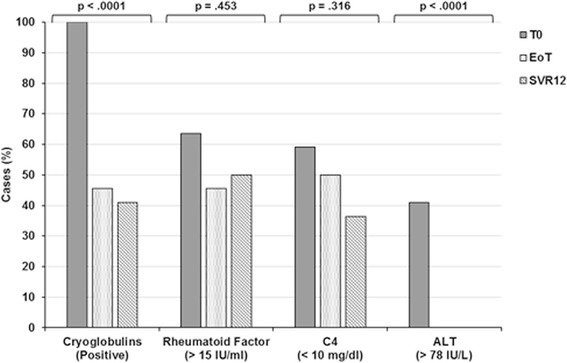
Cross-tab analysis and χ^2^ tests showing modifications of cryocrit, rheumatoid factor (RF), C4 and ALT levels in relation to antiviral therapy. Besides hepatitis C virus RNA, cryocrit and ALT values were significantly influenced by therapy with respect to RF and C4 (*p* < 0.0001 and *p* < 0.0001, respectively). *ALT* Alanine transaminase, *C4* Complement component C4, *EoT* End of treatment, *SVR12* Sustained virological response 12 weeks after therapy completion

Compared with basal parameters, cryocrit values were significantly decreased at EoT and SVR12 (*p* < 0.0001 and *p* = 0.001, respectively) (Table 2). The decrease in ALT levels (*p* < 0.0001 and *p* < 0.0001, respectively) and the normalisation of C4 levels (*p* = 0.003 and *p* = 0.002, respectively) also reached statistical significance. Conversely, RF activity was found to be significantly reduced at EoT but not at SVR12 (*p* = 0.003 and *p* = 0.083, respectively). The results of univariate analysis were statistically significant for all parameters. In addition, considering the effect size value (η^2^), a remarkable effect of therapy was observed for all biochemical parameters, the effect being particularly prominent for cryocrit and ALT levels (η^2^ = 0.470 and η^2^ = 0.572, respectively). These results were confirmed by cross-tabs and χ^2^ tests, in that the disappearance of cryoglobulins and ALT normalisation were strongly influenced by therapy (Fig. 3).

**Table 2:** Statistical analysis of the changes in the major biochemical parameters

Parameters	Baseline	End of treatment		SVR12		Univariate analysis		
	Mean ± SD (range)	Mean ± SD (range)	*p* Value	Mean ± SD (range)	*p* Value	*p* Value	η^2^	Power
Cryocrit, %	1.8 ± 1.3 (0.5–4)	0.4 ± 0.6 (0–2)	<0.0001	0.8 ± 1.4 (0–4.6)	0.001	<0.0001	0.470	0.999
Rheumatoid factor, IU/L	69.3 ± 119.1 (0–530)	30.9 ± 58.8 (0–255)	0.003	40.1 ± 63.0 (0–244)	0.083	0.002	0.281	0.913
C4, mg/dl	9.6 ± 7.3 (0–24)	13.6 ± 8.8 (4–36)	0.003	14.1 ± 9.4 (2–40)	0.002	0.001	0.313	0.962
ALT, IU/L	104.8 ± 144.7 (13–668)	20.9 ± 5.6 (12–32)	<0.0001	21.1 ± 6.6 (8–32)	<0.0001	<0.0001	0.572	1.000

*Abbreviations: ALT* Alanine transaminase, *C4* Complement component C4, *SVR12* Sustained virological response 12 weeks after therapy completion

In terms of clinical manifestations, we observed regression of Meltzer’s triad of symptoms (cryoglobulinaemic glomerulonephritis and peripheral neuropathy), which either were no longer present or were remarkably reduced in 20 patients (90.9%) and 16 patients (72.7%) at EoT and SVR12, respectively. Two patients with peripheral neuropathy had only a partial benefit during antiviral treatment and showed progressive deterioration after EoT. One patient with glomerulonephritis experienced a worsening of renal function at the time of SVR12, but progression of glomerulonephritis to end-stage kidney disease was not observed. Another patient complained of persistent purpuric rashes throughout the treatment and the follow-up period. The patient with SLL treated with DAAs experience a tumour progression that required chemotherapy with fludarabine and cyclophosphamide in addition to RTX. No recurrence or disease progression was recorded in the two patients with previously treated HCC.

According to the above-mentioned response criteria at the time of EoT and SVR12, a CR was observed in 17 patients (77.3%) and in 14 patients (63.7%), respectively, and a partial virologic plus clinical or immunological response was seen in 4 patients (18.2%) and 5 patients (22.7%), respectively. Also at the time of EoT and SVR12, one patient (4.5%) and three patients (13.6%), respectively, achieved only SVR. At SVR12, a CR was obtained in 8 of 14 patients receiving SOF/RBV, in all 4 patients receiving SOF/LDV, and in 2 of the 3 patients receiving 3D combo therapeutic regimens. At the same time point, a PR was observed in 3 of the 14 patients receiving SOF/RBV, in the only patient receiving SOF/DCV, and in 1 of the 3 patients receiving 3D combo. Three of the fourteen patients who were given SOF/RBV achieved SVR only. Given that all patients reached an SVR 12 weeks after stopping therapy, 86.4% of patients in our series obtained a clinical and/or immunological benefit.

Comparing these results with those of our previous study in which we assessed the response to pIFN-α + RBV + RTX combination therapy [19], we found that the all-oral therapeutic approach was significantly better in terms of CR achievement (*p* = 0.0008 and *p* =0.030 at EoT and SVR12, respectively) (Table 3).

**Table 3:** Response rates in 22 patients with mixed cryoglobulinaemia following treatment with direct-acting antiviral agents at the end of treatment and sustained virological response 12 weeks after therapy

Type of response	pIFN-α/RBV [19], *n* (%)	DAAs therapy, *n* (%)					
		EoT	*p* Value	Power	SVR12	*p* Value	Power
Complete	5 (33.3)	17 (77.3)	0.0008	0.895	14 (63.7)	0.030	0.867
Partial	5 (33.3)	4 (18.2)	0.216	0.801	5 (22.7)	0.553	0.895
SVR only	5 (33.3)	1 (4.5)	0.010	0.926	3 (13.6)	0.102	0.830

*Abbreviations: DAA* Direct-acting antiviral agent, *EoT* End of treatment, *pIFN-α* Pegylated interferon-α, *RBV* Ribavirin, *SVR12* Sustained virological response 12 weeks after therapy completion

Results achieved with pIFN-α/RBV regimen are given by comparison

All patients enrolled in the present study were kept under follow-up, with the aim of assessing the persistence of SVR and the possible changes of clinical and immunological features. At the time of this writing, 20 patients (90.9%) have achieved SVR24. Among them, cryoglobulins reappeared in three patients (including the patient with SLL), despite the persistence of SVR24 and in the absence of clinical relapse. In one patient, the test for cryoglobulins was negative at EoT, was positive again at SVR12 and then disappeared at SVR24. So far, 6 of the 14 CR patients at SVR12 have been followed for 48 weeks after completion of therapy and have maintained a CR. In two of them, cryoglobulins were absent, although RF activity was still present at levels less than baseline. In two additional patients, the cryocrit was reduced at roughly 50% of basal value since EoT and remained unchanged throughout follow-up. In the last two patients, both cryocrit and RF remained undetectable. Moreover, one patient with PR and persistence of symptomatic neuropathy despite undetectable cryoglobulins and two SVR-only responsive patients with symptomatic cryoglobulinaemia reached 48 weeks of follow-up.

No severe adverse events related to the administration of DAAs were observed. Two patients receiving SOF/RBV developed progressive anaemia that required reduction of the RBV dosage and erythropoietin administration. Fatigue and mild pain of the dorsolumbar region were reported by several patients, but in none of them was treatment discontinuation required.

## Discussion

Following the identification of HCV as the largely prevalent etiologic agent of MC, researchers in several studies have evaluated the therapeutic efficacy of antiviral drugs such as pIFN-α, with or without RBV, in this condition [14–16]. These studies consistently reported lower rates of SVR in HCV-positive patients with than in those without MC. In addition, IFN-based regimens were characterised by severe adverse events, often leading to therapy discontinuation. In consideration of the underlying B-cell clonal expansion that characterises MC, B-cell depletion therapy with RTX has also been employed after or in addition to the antiviral treatment [17–19]. MC can indeed be considered as an indolent, benign, lymphoproliferative disease potentially susceptible of evolving into malignant B-NHL [8].

The introduction into clinical practice of the new DAAs for therapy of HCV infection offers for the first time the possibility of achieving SVR in >90%, and virtually all, of the chronically infected patients [21], with the coveted perspective of viral eradication and clinical benefits also in patients with extrahepatic manifestations of HCV infection such as MC.

In this study, we report a single-centre experience with the use of the new DAAs in the therapy of HCV-related MC. The first striking result to be emphasised is the high rate of SVR12. All patients reached this primary endpoint, a dramatic reduction of viraemia starting from the fourth week of therapy. A slightly lower response rate has been demonstrated in patients with MC than in those obtained in patients without cryoglobulinaemia with chronic HCV infection [24, 26]. The suboptimal efficacy of the SOF/RBV combination employed in those studies has been considered as a possible factor accounting for the reduced SVR rates. At variance from these observations, all seven of our patients with genotype 1 HCV infection who were treated with SOF/RBV regimen reached SVR12. Gragnani et al. [26] similarly reported 100% SVR12 and SVR24 by using different SOF-based antiviral combinations, confirming the very high antiviral efficacy of these drugs. More recently, SVR12 was found in 94% of 64 patients with MC treated with DAAs [27].

As reported above, at SVR12, cryoglobulins disappeared or decreased by at least 50% in 77.3% of our patients, whereas RF levels normalised in 50% and C4 in 63.6% of the patients. Furthermore, statistical analysis (i.e., univariate analysis, cross-tabs and χ^2^ test) showed that cryocrit and ALT levels were the biochemical parameters more significantly influenced by therapy. Because ALT is a biochemical marker of virus-induced hepatocytolysis, its normalisation is obviously expected to follow viral load reduction or disappearance. Similarly, because viral particles are constitutive components of the cryoprecipitating immune complexes, the formation of such complexes is clearly affected when viral particles are no longer present following the administration of DAAs.

On one hand, RF and C4 levels are less influenced by DAAs within the short period of 12 weeks after stopping therapy and may represent independent markers not only of clinical activity but also of persistently activated B-cell clones. On the other hand, on the basis of our experience, variable changes of RF and C4 levels may occur when a more prolonged follow-up is taken into consideration, in step with either cryoglobulin disappearance or recurrence.

The administration of DAAs was also shown to result in a remarkable improvement of the clinical manifestations, although symptoms persisted or worsened in six patients (27.3%) despite their achievement of SVR12 or more. Even so, the overall results (63.7% of CR plus 22.7% of PR) were significantly better than those obtained with pIFN-α + RBV therapy [19] or with a triple-combination therapy that included first-generation protease inhibitors [20], with the additional advantage of less frequent and less severe side effects in patients receiving DAAs.

Sollima et al. [32] reported a clinical response at week 12 after treatment in two of seven HCV-positive patients with cryoglobulinaemia who were given all-oral antiviral therapy, but one of them had relapsed vasculitis 8 weeks later. It can be inferred that in some cases the pathogenetic process underlying MC progresses despite viral clearance and that, when a “point of no return” has been overstepped, the immune system keeps synthesising pathogenic virus-independent immune complexes. At the same time, when organ damage appears well established, regression cannot be reasonably expected following antiviral therapy. In our study, only a partial benefit was observed in patients with peripheral neuropathy. Renal function worsened but did not reach end-stage renal disease in one patient with cryoglobulinaemic glomerulonephritis, and vasculitic skin lesions persisted in another patient.

At variance from previous studies in which researchers reported NHL remission following antiviral therapy [33, 34], we observed disease progression in a patient with SLL. Similar observations were reported by Gragnani et al. [26] and more extensively by Arcaini et al. [35], who studied 46 HCV-positive patients with indolent NHL treated with IFN-free antiviral regimens and achieved SVR12 in 98% of the patients and lymphoproliferative disease response of splenic marginal zone lymphomas in 73% of the patients. On the contrary, NR was reported in patients with chronic lymphocytic leukaemia/SLL. Possible explanations for these findings are that DAAs lack the anti-proliferative and immunomodulatory effects of IFN-α and that chronic HCV infection has a different impact on the oncogenic process in this particular subset of NHLs.

Interestingly, new clinical scenarios are being opened following the treatment with DAAs of patients with chronic HCV infection who have hematologic malignancies [36]. In addition to preventing the development of some NHLs, the use of DAAs in fact may avoid HCV reactivation and hepatic flare after chemotherapy, may induce a better outcome after allogeneic bone marrow transplantation, and may reduce the risk of hepatic failure due to cytotoxic drugs, especially in patients with liver cirrhosis [36].

## Conclusions

Although the worldwide experience with the use of DAAs in HCV-positive patients with cryoglobulinaemia is still limited, our single-centre study shows that variable combinations of DAAs are able to induce SVR12 and SVR24 in virtually all patients. At 12 weeks after EoT, CR and PR were diagnosed in as many as 86.4% of the patients. These results not only are strikingly better than those obtained with previous antiviral treatments [14–16, 20, 37] but also are closely comparable, in terms of SVR12, to the results reported in remarkably more numerous multicentre cohorts of patients with chronic HCV infection without MC [22, 23].

However, in step with the remarkably high rates of virus eradication induced by the growing cluster of DAAs, new and unforeseen scenarios are emerging. In a minority of patients, clinical and immunological features of cryoglobulinaemic vasculitis persist despite viral clearance. The long-term evolution of these features is totally unknown, and whether an early antiviral approach with DAAs, before the occurrence of severe organ damage, might be able to prevent the occurrence of these initially virus-induced and then virus-cleared vasculitides remains to be elucidated. In addition, at variance from patients with MC achieving SVR and a successful vasculitic response, in whom the regression of B-cell monoclonal expansion in the bone marrow has been described [19], the presence of circulating RF may be associated with the over-representation of mature activated memory B cells, which persist at least during the early phase following eradication of HCV [38]. To ascertain whether an HCV-independent, B-cell lymphoproliferative disorder may eventually appear in this particular group of patients, more prolonged follow-up after viral clearance is necessary.

## Abbreviations

3D combo: Ombitasvir/paritaprevir/ritonavir plus dasabuvir
AIFA: Italian Medicines Agency
ALT: Alanine transaminase
B-NHL: B-cell non-Hodgkin lymphoma
CR: Complete response
DAA: Direct-acting antiviral agent
DCV: Daclatasvir
EoT: End of treatment
GLM: General linear model
GT: Genotype
HCC: Hepatocellular carcinoma
HCV: Hepatitis C virus
IFN: Interferon
Ig: Immunoglobulin
LDV: Ledipasvir
MC: Mixed cryoglobulinaemia
NHL: Non-Hodgkin lymphoma
NR: No response
pIFN: Pegylated interferon
PR: Partial response
RBV: Ribavirin
RF: Rheumatoid factor
RTX: Rituximab
SLL: Small lymphocytic lymphoma
SOF: Sofosbuvir
SVR: Sustained virological response
SVR12: Sustained virological response 12 weeks after therapy completion
SVR24: Sustained virological response 24 weeks after therapy completion
